# Association between tooth loss and geriatric syndromes in older adults from a rural area in eastern China

**DOI:** 10.1093/geroni/igaf122.2430

**Published:** 2025-12-31

**Authors:** Jing Chen, Caihong He, Qin Zhang

**Affiliations:** The First Affiliated Hospital, Zhejiang University School of Medicine, Hangzhou, Zhejiang, China (People’s Republic); The First Affiliated Hospital, School of Medicine, Zhejiang University, Hangzhou, Zhejiang, China (People’s Republic); The First Affiliated Hospital, Zhejiang University School of Medicine, Hangzhou, Zhejiang, China (People’s Republic)

## Abstract

We aimed to explore the associations between tooth loss, denture status, and geriatric syndromes.In 2019, 1094 older adults from a rural area in eastern China were enrolled and a sub-sample of the participants (N = 690) were followed up in 2023. Logistic regression analysis was utilized to explore the association between tooth loss severity at baseline and geriatric syndromes at fourth year follow-up. The multivariable models showed that compared to those without tooth loss or with tooth loss not affecting daily life, those having tooth loss affecting daily life at baseline was associated with a 1.80-fold higher odds of sarcopenia (95% CI: 1.02 to 3.21) and a 2.31-fold higher odds of malnutrition risk (95% CI: 1.44 to 3.69) at the fourth year. Compared to those with 21 or more teeth, participants with fewer than 10 teeth had 1.87-fold odds for sarcopenia (95% CI: 1.07 to 3.26), 2.99-fold for malnutrition risk (95% CI: 1.93 to 4.62), and 1.68-fold for frailty ( 95% CI: 1.10 to 2.56). Older adults with tooth loss who did not have dentures exhibited a significantly higher risk of sarcopenia, malnutrition risk, frailty, and falls, more number of geriatric syndromes.Our findings suggest that higher severity level of tooth loss at baseline were associated with higher risk of geriatric syndromes at fourth year in older adults. Dentures might mitigate this risk but they do not fully bridge the gap compared to retaining natural teeth. Screening and intervening oral health is important for the prevention of geriatric syndromes in older adults.

